# Metal-Based Complexes in Cancer Treatment

**DOI:** 10.3390/biomedicines10102573

**Published:** 2022-10-14

**Authors:** Tania Gamberi, Muhammad Hanif

**Affiliations:** 1 Department of Experimental and Clinical Biomedical Sciences, University of Florence, Viale Morgagni 50, 50134 Firenze, Italy; 2 School of Chemical Sciences, The University of Auckland, Private Bag 92019, Auckland 1142, New Zealand

Metal-based complexes contribute a vital part to the available arsenal of cytotoxic agents today. Platinum(II) complexes, specifically targeting genomic DNA (e.g., cisplatin, carboplatin, and oxaliplatin), are widely used in the clinic to treat various cancers. Nearly 50% of cancer patients, who undergo chemotherapy, receive a platinum drug either alone or in combination therapy. Despite their central role in cancer chemotherapy, platinum drugs suffer serious drawbacks such as the limited spectrum of antitumor activities, systemic dose-related toxicity, and the frequent induction of drug resistance, often leading to treatment failure. These observations have prompted a strong interest in the investigation of nonplatinum metal-based drugs as an effective alternative. Several other platinum and nonplatinum metal complexes (e.g., Ti, Pd, Ru, Au) have shown potent cytotoxic and antitumor effects. Unlike platinum drugs, these often rely on DNA-independent biochemical mechanisms such as targeting tumor-associated proteins and induction of immunogenic antitumor properties for their therapeutic effects. Notably, a few of these drugs are already in clinical trials, and several are at advance stage of preclinical development.

In this context, the “Metal-Based Complexes in Cancer Treatment” Special Issue was edited, focusing on the design and development of targeted metal-based anticancer agents, understanding their mechanisms of action, and innovative drug delivery approaches. This Editorial briefly summarizes the findings and highlights of the nine published papers (six original research and three reviews).

Among the six original articles, the first published study was by Shakil and colleagues [1]. With the aim of synthesizing novel metal drugs, the authors explored the potential anticancer activity of new hydroxypyridones (HPs) and hydroxy(thio)pyridones (thio-HPs) derivates and their respective ruthenium (Ru) complexes in several human cancer cell lines (colorectal, non-small cell lung, cervical and colon cancer cell lines). They demonstrated the higher antiproliferative activity of the thio-HPs derivates, while the HPs and Ru complexes of both compound types were less potent, despite still showing IC_50_ values in the µM range. The most potent thio-HPs derivates were further tested on other cancer cell lines revealing higher cytotoxicity towards lung cancer cells in comparison to breast cancer cells and human prostate epithelial cells. The biological analysis pointed out the ability of these compounds to trigger apoptotic cell death without affecting cell cycle progression.

In the second published original article by Dorovskikh [2] and colleagues, the noble metals were used to modify the surface of implant materials with the aim of improving their biological properties such as chemical inertia, high biological compatibility, corrosion resistance, and reduced risk of developing toxic or allergic reactions as well as bacterial infections. The authors developed a new approach to modify the surface of implant materials obtaining the most promising results with silver, gold, and platinum coatings.

The study published by Gorini [3], and colleagues dealt with the attempt to improve the knowledge of gold metal-based compounds as anticancer agents. In detail, they deeply investigated the anticancer properties of two dinuclear oxo-bridged gold(III) compounds (Au_2_phen and Auoxo6) against A2780 human ovarian cancer cells. Their findings pointed out that for these compounds a proapoptotic effect similar to other previously characterized gold(III) compounds such as the complex AuL12. The study showed that the apoptotic signal was attributed to the inhibition of the thioredoxin reductase enzyme and the consequent unbalance of the cell redox state, which lead to impairment of the mitochondrial membrane potential, and induction of associated metabolic changes. In addition, evidence was gained of the remarkable contribution of ASK1 (apoptosis-signal-regulating kinase-1) and AKT pathways to gold(III)-induced apoptotic signaling.

Schoch and colleagues investigated the antitumor activity of the experimental platinum(IV)–nitroxyl complex PN149 compound [4], aimed to overcome the severe side effects, such as nephrotoxicity associated with the clinically approved platinum chemotherapeutics. PN149 in comparison with cisplatin, carboplatin, and oxaliplatin was tested on various cancer cells and in the proximal tubule epithelial cell line ciPTEC. The results pointed out a similar cytotoxic behavior for PN149 and the platinum chemotherapeutics. Conversely, PN149 presented a reduced nephrotoxic potential, thus making this compound interesting for further analysis.

Vojtek and co-workers [5] explored, for the first time, the cytotoxicity of the dinuclear palladium(II)-spermine chelate Pd_2_Spm against triple-negative breast cancer (TNBC), a breast carcinoma characterized by low prognosis and limited treatment options based mainly on platinum(II) chemotherapeutics such as cisplatin and carboplatin. The authors demonstrated that Pd_2_Spm owns an anticancer activity in vivo and in vitro similar to platinum derivates but exhibiting lower systemic toxicity, suggesting the potential use of this Pd(II) drug for the treatment of TNBC.

Geyl and colleagues [6], based on the ability of 2-pyridyl urea derivatives to form complexes with transition metals, synthetized 12 new 2-pyridyl urea-based Cu(II) complexes and evaluated their potential anticancer activities against several lung cancer cell lines as well as the healthy lung fibroblast WI-26 VA4 cell line. Two of these complexes showed selective antiproliferative effects against the drug-resistant cell line NCI-N1975 but not toward the non-tumoral cell line suggesting the importance of the ligands in the cytotoxicity of 2-pyridyl urea-based Cu(II) derivates.

Among the three published reviews, Cirri and co-workers [7] addressed four different strategies to improve metal-based cancer treatments. These approaches include (i) drug repurposing, (ii) the chemical modification of approved metal-based drugs, (iii) novel drug combinations and (iv) coupling of newly synthesized complexes to different anticancer drugs.

The review of Babak and co-workers [8] dealt with the potential anticancer strategies based on the modulation of intracellular copper levels. They presented strategies to achieve this unbalance of intracellular copper levels and the subsequent toxicity in cancer cells. These approaches include: (i) modulation of Cu levels with metal-binding ligands such as Cu chelators and Cu ionophores, (ii) modulation of Cu levels with pre-formed Cu complexes.

Finally, McGhie and colleagues [9] reviewed the innovations in metal-based anticancer therapies during the last ten years based on transition metal complexes with photoactive or luminescent properties. They reported examples where luminescent complexes are employed to induce the anticancer effects through a photodynamic therapy approach (PDT) based on photosensitizers, light, and oxygen to damage cancer cells, and through photoactivated chemotherapy (PACT), where light activates a prodrug.

The issue brings together a wide variety of articles about metal-based anticancer agents. It has been our pleasure to edit these exciting articles. The editors hope the papers will interest researchers in the field of anticancer drug discovery. The articles will improve the understanding of the properties of anticancer metal complexes for future drug design and discovery.
